# Subtelomeres are fast-evolving regions of the *Streptomyces* linear chromosome

**DOI:** 10.1099/mgen.0.000525

**Published:** 2021-03-22

**Authors:** Jean-Noël Lorenzi, Olivier Lespinet, Pierre Leblond, Annabelle Thibessard

**Affiliations:** ^1^​ Université Paris-Saclay, CEA, CNRS, Institute for Integrative Biology of the Cell (I2BC), 91198, Gif-sur-Yvette, France; ^2^​ Université de Lorraine, INRAE, DynAMic, F-54000 Nancy, France

**Keywords:** *Streptomyces*, linear chromosome, recombination, horizontal gene transfer

## Abstract

*
Streptomyces* possess a large linear chromosome (6–12 Mb) consisting of a conserved central region flanked by variable arms covering several megabases. In order to study the evolution of the chromosome across evolutionary times, a representative panel of *Streptomyces* strains and species (125) whose chromosomes are completely sequenced and assembled was selected. The pan-genome of the genus was modelled and shown to be open with a core-genome reaching 1018 genes. The evolution of *Streptomyces* chromosome was analysed by carrying out pairwise comparisons, and by monitoring indexes measuring the conservation of genes (presence/absence) and their synteny along the chromosome. Using the phylogenetic depth offered by the chosen panel, it was possible to infer that within the central region of the chromosome, the core-genes form a highly conserved organization, which can reveal the existence of an ancestral chromosomal skeleton. Conversely, the chromosomal arms, enriched in variable genes evolved faster than the central region under the combined effect of rearrangements and addition of new information from horizontal gene transfer. The genes hosted in these regions may be localized there because of the adaptive advantage that their rapid evolution may confer. We speculate that (i) within a bacterial population, the variability of these genes may contribute to the establishment of social characters by the production of ‘public goods’ (ii) at the evolutionary scale, this variability contributes to the diversification of the genetic pool of the bacteria.

## Data Summary

In this work, we used 125 chromosomal sequences deposited in NCBI database. Their accession numbers are listed in Table S1 (available in the online version of this article)

**Impact Statement**

*
Streptomyces* are bacteria that, unlike most others, have a large and linear chromosome that consists of a central region flanked by two variable arms. Our work aims at understanding the evolutionary forces that are imposed on this chromosome and have shaped this compartmentalization over evolutionary times. For that purpose, we established a set of 125 *Streptomyces* chromosomes from public databases and monitored indexes measuring the conservation of genes (presence/absence) and their organization (synteny) along the chromosome. Using the phylogenetic depth offered by the chosen panel, it was possible to infer that within the central region of the chromosome, the core-genes form a highly conserved organization, which can reveal the existence of an ancestral chromosomal skeleton. Conversely, the chromosomal arms, enriched in variable genes evolved faster than the central region under the combined effect of rearrangements and addition of new information from horizontal gene transfer. Such an ability to diversify the genome content could contribute to *Streptomyces* adaptability to the soil, which is a naturally challenging ecosystem.

## Introduction

Horizontal gene transfer (HGT) is the most efficient way to diversify the genetic pool of a group of bacterial individuals, whatever the phylogenetic level considered, i.e. population, species or genus [1]. It allows the acquisition in a single event of ‘ready to use’ genes and contributes to the extension of the accessory genome, which are the genes present only in a fraction of the strains. It provides a means of rapid adaptation to environmental changes by keeping a strong diversity within a population from which adapted individuals can emerge. It can also provide specific advantages to the whole population in nature, some individuals being able to produce compounds in the micro-environment (e.g. antibiotics) for the benefit of the whole group [2]. Therefore, the accessory gene pool can be considered as public goods [3]. The pan-genome defined as the entire and non-redundant gene set of the group is being said to be open when it continuously increases with the number of analysed genomes [4]. Reciprocally, when very few genes are added per sequenced genome, the pan-genome is said to be closed. Open pan-genomes are notably observed in the case of bacteria that present an autonomous lifestyle and are involved in biotic interactions and therefore exposed to exogenous information [5]. Hence, HGT firstly requires the internalization of the exogenous genetic material [6]; this step depends upon three main mechanisms known as conjugation, transduction and natural transformation. Secondly, this incoming DNA in the new host has to be maintained. This maintenance relies either (i) on the recipient recombination abilities (homologous and illegitimate recombination pathways, thereafter abbreviated by HR and IR, respectively) or (ii) on mechanisms encoded by the mobile genetic elements (MGEs) involved in HGT promotion themselves [e.g. replication of plasmids, integration of integrative and conjugative elements (ICEs) or phages…). Endogenous recombination mechanisms can indeed lead to allele replacement or to addition of new sequences in the host genome. Allele replacement results from HR between highly similar gene sequences (it remains undetected if recombination occurs between identical sequences [7]), addition of new sequences can result from integration of new information by either HR or IR [8, 9]. Although HR is favoured by the genetic relatedness between the partners, IR would not be subordinate to sequence homology between the incoming DNA and the recipient chromosome. At last, the long-term maintenance of a DNA sequence depends on its impact on bacterial fitness [10].

*Streptomyces* are prominent bacteria found in soil and marine environments [11]. They are committed in complex biotic interactions with bacteria, fungi, plants and insects [12] and involved in biogeochemical recycling thanks to their prolific specialized metabolism (chemical compounds and enzymes). Diversity of the *Streptomyces* genus was empirically exploited for decades for the production of antibiotics, antifungal and other metabolites of medical or biotechnological interest; each species was famous for the production of a few compounds [13].

*Streptomyces* harbour a linear chromosome (6–12 Mb) [14] ended by terminal inverted repeats (TIRs) of various sizes reaching up to several hundreds of kilobases [15]. Genome comparison within a growing set of *Streptomyces* sequenced genomes showed that their linear chromosome is typified by a very peculiar organization: most of the genes shared by all the considered species (core-genes) are localized in the central part of the chromosome while the chromosomal arms carry more variable genes [14, 16–18]. This compartmentalization may find its origin in the linearization process assumed to have accompanied the emergence of the *Streptomyces* genus [18]: in a parsimonious hypothesis, acquisition of telomeres and of chromosomal arms would have resulted from a single integration event of a linear replicon within the ancestral circular chromosome. Consequently, chromosomal arms would have included contingent genes since this early linearization event. This compartmentalization corroborates early studies on genetic instability of *Streptomyces* species [15, 19] showing that subtelomere regions are, in lab conditions, prone to huge DNA rearrangements (deletion, amplification,…) of over several hundreds of kilobases, presumably because of the lowering of selective pressure exerted on the contingent genes present in these regions (extending over up to several tens of percent of the genome). In an extensive genome analysis of 80 bacterial species, Oliveira *et al*. [20] found that, within a given species, transferred genes are concentrated in only ~1 % of the chromosome and formed hotspots.

These hotspots can be associated with MGEs, notably ICEs. When no trace of MGEs is associated with HGT hotspots, integration was found to result from HR using flanking conserved genes or sequences [20]. In *Streptomyces *, although the genome is rich in (i) conjugative elements, ICEs depending on a type IV secretion system and (ii) actinomycete ICEs (AICEs) whose transfer depends on a DNA translocase (TraB) [21, 22], there is no evidence of a direct link between these MGEs and the dynamics of the chromosome. However, since AICEs are capable of mobilizing chromosomal DNA in trans, i.e. not physically linked to the element [23], it can be hypothesized that gene fluxes are driven by conjugational processes. In a previous study, we have shown that early diversification events between closely related strains consisted in insertions and deletions preferentially occurring in the chromosomal arms [24]; in this work, we exploited the phylogenetic depth of the whole *Streptomyces* genus to acquire a dynamic picture of its evolution. We questioned the basis of the genome diversification in *Streptomyces *. How do the different regions of the chromosome evolve (central part versus arms)? Are the chromosomal arms more tolerant to DNA rearrangements and do they constitute fast-evolving regions?

## Methods

### Average nucleotide identity (ANIb) computation

The average nucleotide identity (ANI) between query and reference genomes was calculated by using the blastn algorithm, ANIb [25]. In a first step, the query genome was fragmented into 1000 consecutive parts, each of them was then aligned to the sequence of the reference genome using blastn (v2.2.31+) [26]. The ANIb score corresponds to the average value of the percentages of nucleotide identity of the query fragments having a positive match with the reference genome (alignment greater than 70 % with at least 30 % of nucleotide identity, 25). As the ANIb score is not reciprocal (i.e. the ANIb score of genome A versus genome B may be slightly different from the ANIb score of genome B versus genome A), we used the average of the two reciprocal values as the final score.

### Constitution of a set of genomes representative of *Streptomyces* genus

The 234 complete *Streptomyces* genomes available in the NCBI database [27] as of 17 January 2020 constituted our basic genomic data set. First, the completeness of the assembly of the genomes retained in the dataset was assessed using BUSCO [28] with the streptomycetales dataset (odb10). Second, to avoid redundancy, the genomes were clustered using their ANIb value and genomes sharing a value greater than or equal to 96 % were considered to represent strains belonging to the same species [29]; therefore only one of them was retained in the final dataset. As an exception, *Streptomyces ambofaciens* DSM 40697 and ATCC 23877 were both kept in our dataset as they represent the model species of our laboratory, and allow a comparison of closely related strains. In total, the complete genomes of 125 strains or species were collected (Table S1). All these 125 genomes selected and analysed further in this study are in a single contig.

### Terminal inverted repeats detection and identification

Terminal inverted repeats (TIRs) are defined as perfect repeats of DNA at the ends of chromosomes or linear plasmids. However, these repeats may or may not be present in the assembly published in the database depending of their size, which can vary widely (from a few tenths of nucleotides to several hundreds of kilobases) and the sequencing technologies used (i.e. sequencing from recombinant BACs or cosmids, whole-genome sequencing using long or short reads). The longer the repetition is, the lower is the chance of identifying it as a single piece in a sequence scaffold. To detect the presence of TIRs and to identify their size, we searched for DNA repeats at the ends of all the considered chromosomes. To do this, by using blast, we searched for an alignment between the two chromosomal sequence ends (blastn v2.2.31+, 26). When a repeat was detected (99 % of nucleotide identity), we deduced the TIR length from the position within the sequence (Table S1). In case of identification, only one single copy of the TIRs was kept in the final chromosome sequence.

### Genome annotation, orthology assignment

All genome sequences were automatically annotated on the rast Server [30] using the rast Classic pipeline (FIGfam version: release 70) to provide a homogenous annotation process, and fed the orthology assignment process with a valid and consistent annotation dataset. For each pair of genomes, orthologues were defined by identifying blastp reciprocal best hits (BBH) [31, 32] with at least 40 % of identity, 70 % of coverage and an E-value lower than 1e^−10^.

### Core- and pan-genome computation

The core-genome was determined as the set of orthologues present in all the genomes of a given dataset, and the pan-genome as the sum of the core-genome plus the specific genes found in the genomes of the same dataset. Both growing sets were computed for genome sets ranging from 2 to 125 genomes. For any set of size *n* smaller than 125, core- and pan-genomes were computed by performing 100 iterations of a random selection of *n* different genomes from the 125 genomes available in our dataset. The core- and pan-genomes size for each dataset size can be represented as a box-and-whisker plot with the lower and upper whiskers corresponding to the first and the last quartile of the distribution. Based on the position of the genes belonging to the core-genome, the core-region has been defined as the area including 95 % of them to avoid overestimation of the extent of the core-region (as illustrated for *S. ambofaciens* ATCC 23877 genome in Fig. S1.

### Phylogenetic inference based on maximum-likelihood

For each genome, the protein sequence of the 1018 genes of the core-genome were retrieved. After alignment using muscle v3.8.31 [33] and trimming by Gblocks to eliminate poorly aligned positions [34], the 1018 multiple sequence alignments were concatenated into a unique multiple sequence alignment comprising 324 451 amino acid positions. The multiple alignment was then submitted to RAxML [35] with the LG substitution model for maximum-likelihood-based tree inference. One hundred bootstrap replications were performed. The phylogenetic tree was represented using the Dendroscope software [36].

### Orthology rate (OR), gene order conservation (GOC) and neighbour orthologue conservation (NOC) indexes

The GOC was calculated according to Rocha [37] and reflects the conservation of vertically inherited genes, that is to say the ancestral chromosomal architecture. A high GOC value means a strong conservation of gene organization, a low value reveals the shuffling of the orthologues along the genome, which means that recombination broke the ancestral synteny. This value is calculated by dividing the number of orthologues involved in contiguous pairs between two compared chromosomes by the number of orthologues in the window. A sliding window (1 or 5 % of the total number of genes of the reference genome) was moved along the reference chromosome with a one-gene step to get local values. The OR, on the other hand, gives the proportion of genes of the reference genome that have an orthologue in the compared genome. This index was calculated locally, in a window sliding (one-gene step) along the reference genome in a pairwise comparison. It allows us to measure the influence of the acquired/lost genes in the conservation of the genome architecture. Finally, the NOC was defined and consists in the ratio of the number of orthologues involved in contiguous pairs in the reference strain that are also contiguous in the compared strain. This index used a sliding window (1 or 5 % of the number of orthologues involved in contiguous pairs in the reference) moving along the reference chromosome with a step of 0.1 % of the considered genes. It allows the same number of windows to be had for each genome pairwise comparison with the same reference genome.

### Double-cut and Join (DCJ)

The DCJ model is a genomic rearrangement model used to define an editing distance between genomes based on the gene order and orientation [38]. Rearrangement events that occur in genomes, such as inversions, translocations, fusions and fissions can be represented. In our case, a variant of the DCJ model was used, the restricted DCJ model [39]. The application of the DCJ algorithm requires that the two genomes being compared have the same gene content. Therefore, this method can only be used on orthologous genes for a given pair of species. In order to compare the DCJ distance obtained from different pairs of species, the DCJ distance was divided by the number of orthologous genes involved in the calculation.

## Results

### The pan- and core-genomes reflect a great diversity of gene content in the *Streptomyces* genus

Based on a representative collection of 125 *Streptomyces* genomes (Table S1) (selected from the 234 were available on 17 January 2020), we addressed the question of the global chromosome dynamics inside *Streptomyces* genus. Among our set of 125 species, the genome size can vary in the ratio of one to two, with respectively 6.33 Mb for *Streptomyces seoulensis* KCTC 9819 and 12.00 Mb for *Streptomyces* sp. S1A1 7 (Table S1). At the same time the estimated gene content, based on the results of the rast gene predictor [30], can vary from 5866 genes for *S. seoulensis* KCTC 9819 to 11 068 genes for *Streptomyces* sp. S1A1 7. Between these two extreme values, the distribution of the gene content of the 125 genomes studied here is quite variable with a median of 7350 genes (Fig. S2). This great variability in gene content at the whole genus scale is also attested by the size of the pan-genome (set of unique genes of a given group of bacteria) built from this species set. Actually, the pan-genome of the 125 species appears to be extremely open with more than 106 000 genes (open feature confirmed by Heap’s law constants, Fig. S3): the curve obtained for the pan-genome is far from reaching an asymptote and it is expected that this value will further increase by adding new genomic data. On the other hand, we observed that the size of the core-genome ended up at 1018 genes (Fig. S3), which represents about a tenth of the gene-content median value. This core-genome size is lower than those obtained in previous studies [14, 40], but remains consistent with them, considering the larger number of genomes included in our study. Fig. S3 represents the spectrum of frequencies for *Streptomyces* gene repertoires and shows that 68 % of the pan-genome is composed of specific genes.

The amino acid sequences deduced from the nucleic sequences of the 1018 core-genes were used to perform a multilocus phylogenetic analysis (see Methods). The reconstructed tree (Fig. S4) was in accordance with that previously proposed by McDonald and Currie [41], who suggested that the *Streptomyces* genus can be divided into two major monophyletic clades (clade I and clade II) and a third group of other *Streptomyces* lineages (referred to as ‘others’).

### Distribution of the core-genes along the chromosome

**Fig. 1.**
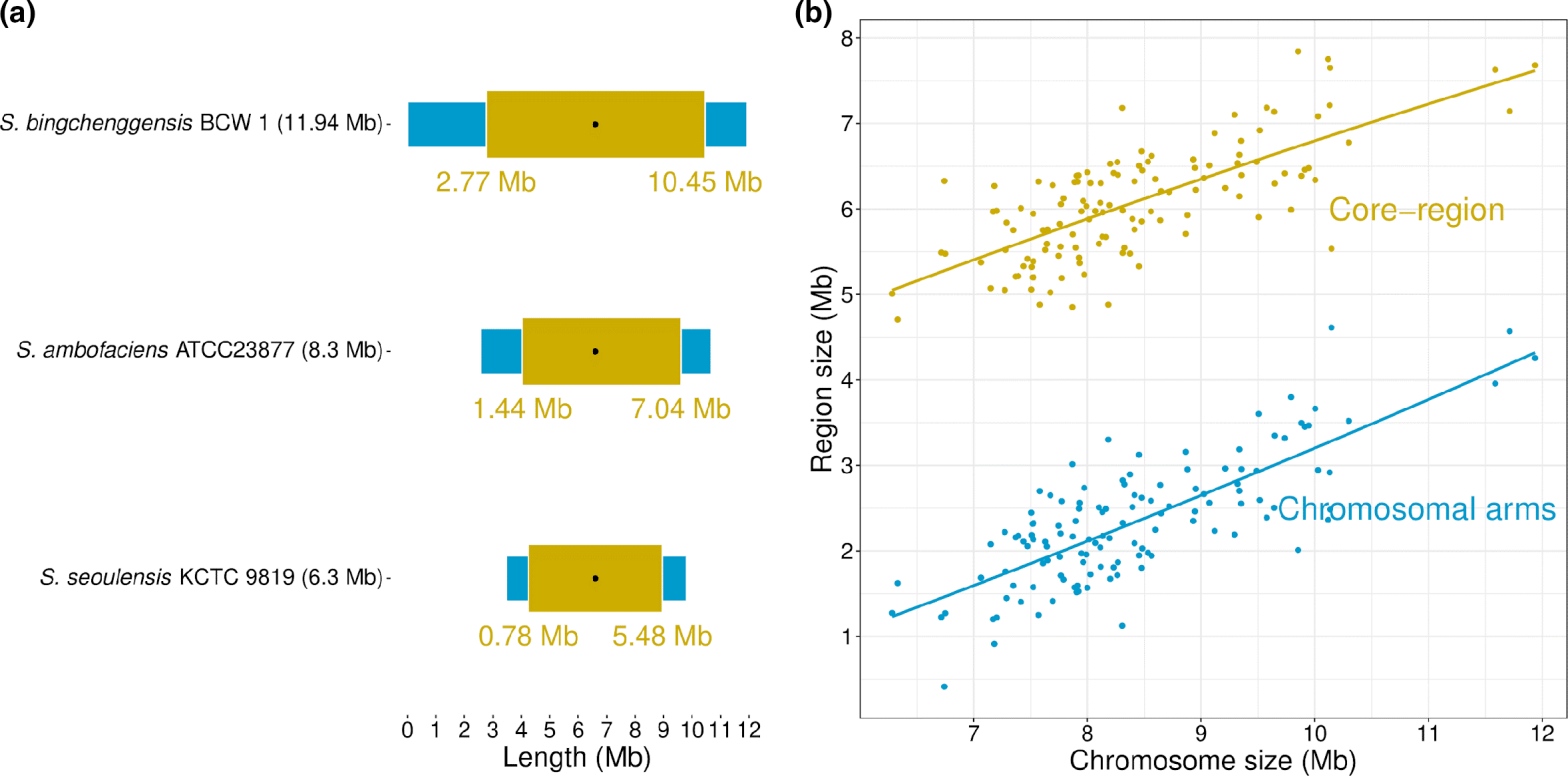
Core-genome and chromosomal arms across *Streptomyces* species. (a) Schematic representation of the compartmentalization of ‘typical’ *Streptomyces* chromosomes. The core-region, defined as the chromosomal area that includes 95 % of the core-genes, is in ochre and the chromosome arms are in blue. The *oriC* locus is symbolized by a black dot. Numbers (Mb) represent the limits of the core-region. (b) Size of the chromosomal arms (cumulated size) and core-genome in the 125 *Streptomyces* species.

For all the species, the 1018 genes of the core-genome were all located on the chromosome. Their distribution along the linear chromosome generally showed a localization limited to the central part (illustrated for *S. ambofaciens* ATCC 23877 in Fig. S1.) defining a ‘core-region’ (i.e. region containing 95 % of the core-genes, see Methods). The flanking regions were called chromosomal arms. In Fig. 1a the chromosomal location of the core-region for three species with different chromosome sizes is illustrated: *S. seoulensis* KCTC 9819 6.33 Mb, *S. ambofaciens* ATCC 23877, 8.3 Mb and *Streptomyces* sp. S1A1-7, 12.00 Mb. The core-regions spread over 4.7, 5.6 and 7.15 Mb, respectively. This survey was extended to the whole-genome set and revealed that most of the chromosomes (99 out of 125) displayed a core-region roughly centred in the chromosome; these chromosomes will later be referred to as the ‘typical architecture chromosomes’. The remaining genomes harboured a core-region that is not central, resulting in unbalanced arms (arbitrarily defined as chromosomes that display one arm twice longer than the other; 26/125). Further, by plotting the size of the core-region and that of the chromosomal arms as a function of the chromosome size (Fig. 1b), it appeared that the size of the arms increased parallel to that of the core-region as a function of the chromosome size. While the core-region size ranged from 4.71 to 7.84 Mb, the chromosomal arm size (sum of the two arm sizes) ranged from 0.41 to 4.61 Mb. Thus, the size of the genome increases by the increase in the size of the arms and by the increase in the size of the core-region. In other words, the expansion of the *Streptomyces* genome would depend on the acquisition of accessory genes in the chromosomal arms as well as within the central region of the chromosome. It can be specified however that the proportion occupied by the core-region tended to decrease slightly (and that occupied by the arms to slightly increase) as a function of the chromosome size.

### Evolution of the genome plasticity along *Streptomyces* chromosome

**Fig. 2.**
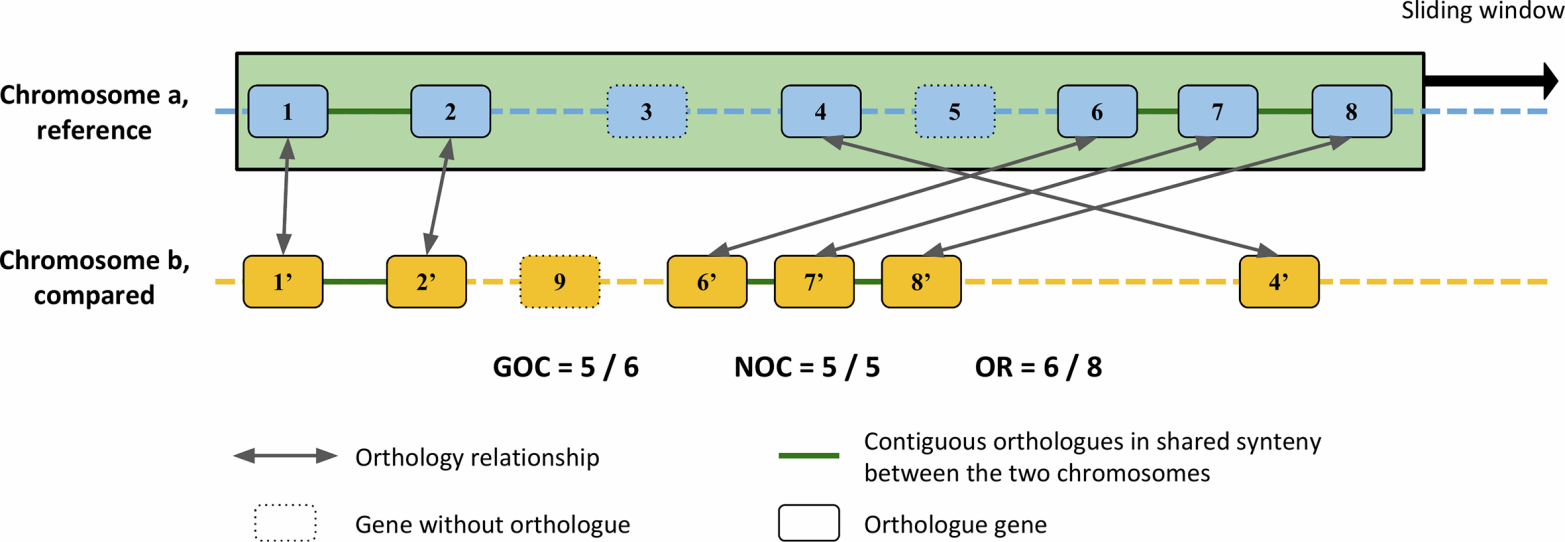
Definition of indexes for genome conservation analyses. Pairwise comparisons are achieved by comparing a reference strain (species a) to another (species b) using a sliding window. A theoretical sliding window containing eight genes is shown on the reference species. For both the GOC and NOC indexes, orthologues of the eight genes are searched for and counted if they also form contiguous pairs in the genome of species b. For the GOC index, this number is normalized by the number of genes among the eight that have an orthologue in genome b. For the NOC index, this same number is normalized by the number of genes that have an orthologue in genome b and belong, in chromosome a, to a group of contiguous genes. In other words, in the second index, genes that have an orthologue in chromosome b but whose neighbours in a (on either sides) have no orthologues in b (i.e. isolated orthologe, as gene 5 of chromosome a) are not considered. The OR index corresponds to the ratio of genes in the window of the reference that have an orthologue in genome b.

In order to identify the origins of the variability of *Streptomyces* chromosome, we assessed the relative impact, on the one hand, of the shuffling of the ancestral information, and on the other hand, of gene flux by loss, acquisition or replacement. For that purpose, several indicators were monitored in pairwise comparisons of genomes of more or less closely related strains (Fig. 2). All these indicators take into account the number of orthologue genes and/or their relative positions in the two compared genomes.

**Fig. 3.**
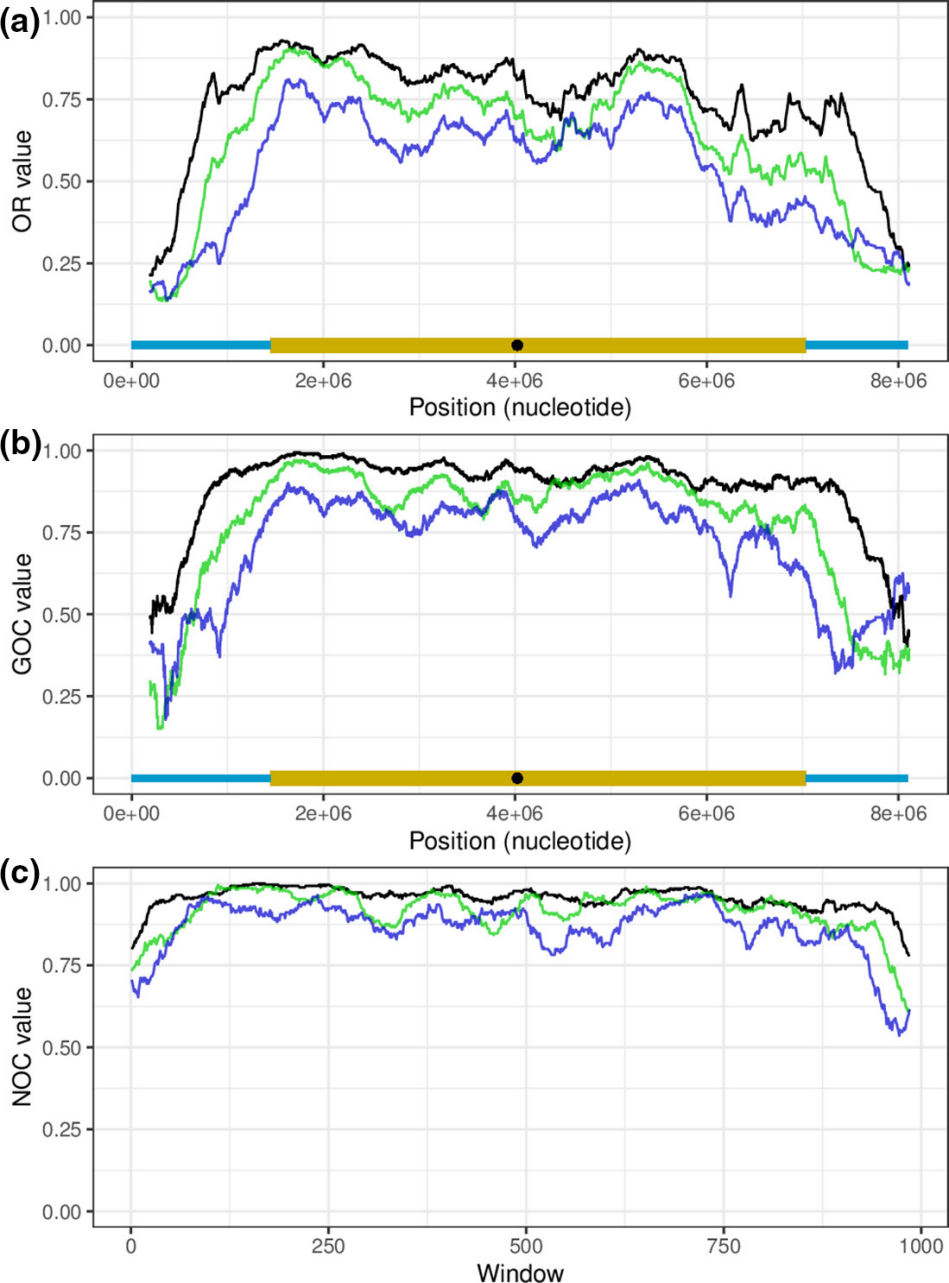
OR, GOC and NOC profiles along the *Streptomyces* chromosome. Profiles are obtained by plotting OR (a), GOC (b) and NOC (c) values obtained by pairwise comparison of *S. ambofaciens* ATCC 23877 as a reference with three species showing tight [* S. coelicolor* A3(2), black curve], average (* S. lincolnensis* LC-G, green curve) or weak (* S. albus* ZD11, blue curve) phylogenetic distances. GOC and OR values were calculated using a sliding window (one-gene step) of size equal to 5 % of the chromosomal gene content of *S. ambofaciens* ATCC 23877. Each window is located on the chromosome by the position of its central gene. NOC values were calculated using a sliding window of size equal to 5 % of the number of genes belonging to groups of contiguous orthologues in *S. ambofaciens* ATCC 23877. This window is sliding along the reference chromosome by a step corresponding to 0.1 % of the considered genes. The NOC values are not related to physical positions, so they are plotted without chromosomal anchoring.

The OR, defined as the rate of genes conserved in both compared genomes whatever their location, was used to estimate the impact of gene fluxes. High OR values indicate a strong gene content conservation while low values reveal a high proportion of specific genes (either lost by one strain or acquired through horizontal transfer by the other). Depending on the relationship between the two compared genomes this number varied from 0.30 to 0.93. At the intra-clade level, the OR values reached a minimum of 0.41 (clade I) and 0.36 (clade II) underlining the intensity of gene fluxes even within a monophyletic clade. In order to visualize a possible difference depending on the chromosomal location, this analysis was conducted locally in a window sliding along the chromosome (see Methods). The result obtained for *S. ambofaciens* as a reference in three pairwise comparisons with species belonging to clade II and sharing various phylogenetic distances [* S. coelicolor* A3(2), *S. lincolnensis* LC-G, *S. albus* ZD11] is shown in Fig. 3a. *S. ambofaciens* ATCC 23877 was chosen as the reference since it is our lab model and belongs to a species cluster including the model *Streptomyces coelicolor* A3(2). As expected, the level of orthology follows the phylogenetic distance: the more two strains were related, the higher the OR value was. Further, there is a strong and progressive decline in the chromosomal arms. This showed that the chromosomal arms accumulate specific genes more intensely than the central part of the chromosome.

**Fig. 4.**
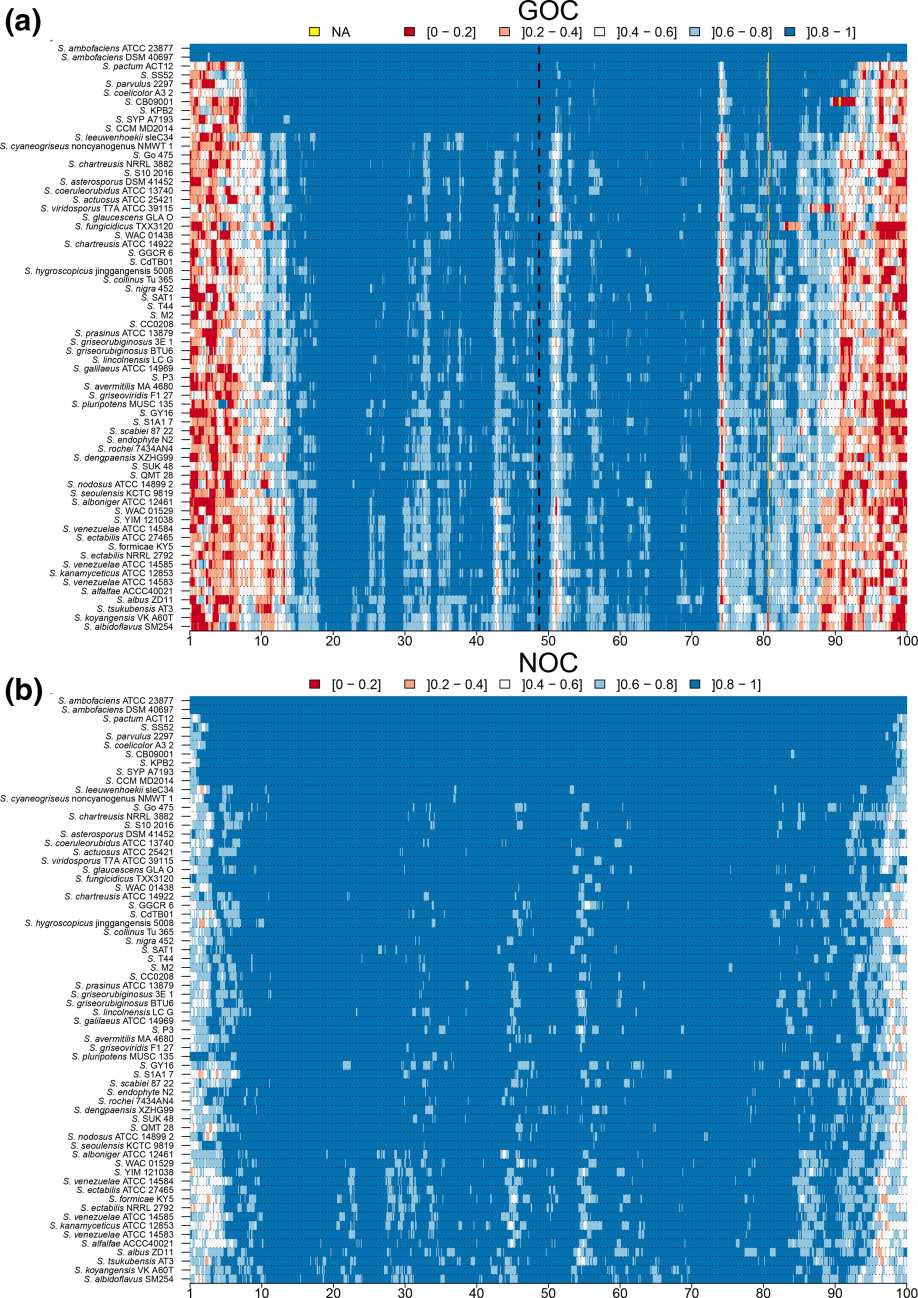
GOC and NOC across clade II species. The GOC trend was monitored along the genome in pairwise comparisons between a reference strain [herein *S. ambofaciens* ATCC 23877, using a sliding window (one-gene step) of size equal to 1 % of the chromosomal gene content] and the genomes of clade II. The profiles were ordered from the closest to the farthest species relative to *S. ambofaciens* ATCC 23877, by using the cophenetic distances computed from the phylogenetic tree. The abscissa axis is expressed as the percentage of the reference chromosome. Each of the percentiles is coloured according to a colour code from red to blue reflecting increasing GOC values. na stands for non-applicable.

GOC as previously defined by Choulet *et al*. [17] and a GOC-derived parameter called NOC for neighbour orthologue conservation were monitored to assess genome shuffling; GOC has been calculated by dividing the number of orthologues involved in contiguous pairs between two compared chromosomes by the number of orthologues in the window (Fig. 2). As for the OR index, the local GOC and NOC indexes were surveyed within a sliding window. When considering the GOC parameter at the local scale, it should be noticed that the particular organization of *Streptomyces* chromosome could give rise to two pitfalls: first, since orthologues are scarce in some chromosomal areas when considering distant species (notably in the arms), some windows could be devoid of orthologues, making the calculation inapplicable (Fig. 4a); second, because when the orthologues were scattered, the probability to have a neighbour that is also an orthologue strongly drops, therefore the number of contiguous orthologous genes in a given window may not be significant. Consequently, these results must be interpreted with caution, especially as the phylogenetic distance increases.

In order to overcome these limitations, the NOC was defined: it consists in the ratio of the number of orthologues involved in contiguous pairs in the reference strain that are also contiguous in the compared strain (Fig. 2). In contrast with GOC, the NOC index can be applied and will be valid whatever the orthology level between the compared genomes. However, NOC windows, defined as 5 % of the total number of orthologous genes organized as contiguous pairs in the considered pairwise comparison, will cover regions of highly different physical sizes. In other words, a window in a poorly conserved region will extend over a larger chromosomal area than a window in a more conserved region. Further, depending on the phylogenetic distance between the compared strains, a common chromosomal locus will be included in windows of different sizes. As a consequence, the trend of the NOC cannot be plotted onto a physical map of the reference strain and therefore cannot be compared to the OR and GOC trends.

Finally, both GOC and NOC indexes turned out to be complementary and relevant to assess genome plasticity across evolutionary times. GOC and NOC analyses were performed for all of the 15 500 pairwise comparisons of species of our dataset; each genome chosen as a reference has been compared to the 124 other genomes. Because of the limitation and cannot be plotted onto a physical map of the reference strain, we chose to restrict the presentation to intra-clade pairwise comparisons. As an example, the results obtained for *S. ambofaciens* ATCC 23877 (clade II) and *S. griseus* NBRC 13350 (clade I) used as a reference are presented as heatmaps, respectively in Figs 4 and S5. When considering ‘typical’ chromosomes, it appeared that the chromosomal arms were much more shuffled than the central part. Further, the synteny break frequency increased together with the phylogenetic distance, demonstrating the cumulative nature of these events. Besides, it seems that, the more the chromosomes compared belong to distant strains, the more the shuffled loci concern more internal regions within the chromosomal arms. This observation could be interpreted as a frequency of rearrangement increasing towards the ends of the chromosome, confirming the notion of a gradient of loss of synteny (called degenerated synteny), proposed by Choulet *et al*. [17]. The NOC index (assessing the conservation of adjacent orthologous genes) showed a similar trend as the GOC and confirmed that the arms are recombinogenic regions. The GOC and NOC results obtained for *S. ambofaciens* as a reference in the pairwise comparisons with the three species *S. coelicolor* A3(2), *S. lincolnensis* LC-g, *S. albus* ZD11 is shown Fig. 3b, c. This focus, plotted as curves, allowed visualization of the progressive decline of both these indexes within the arms when getting closer to the chromosomal ends.

### Detection of a conserved chromosomal skeleton

**Fig. 5.**
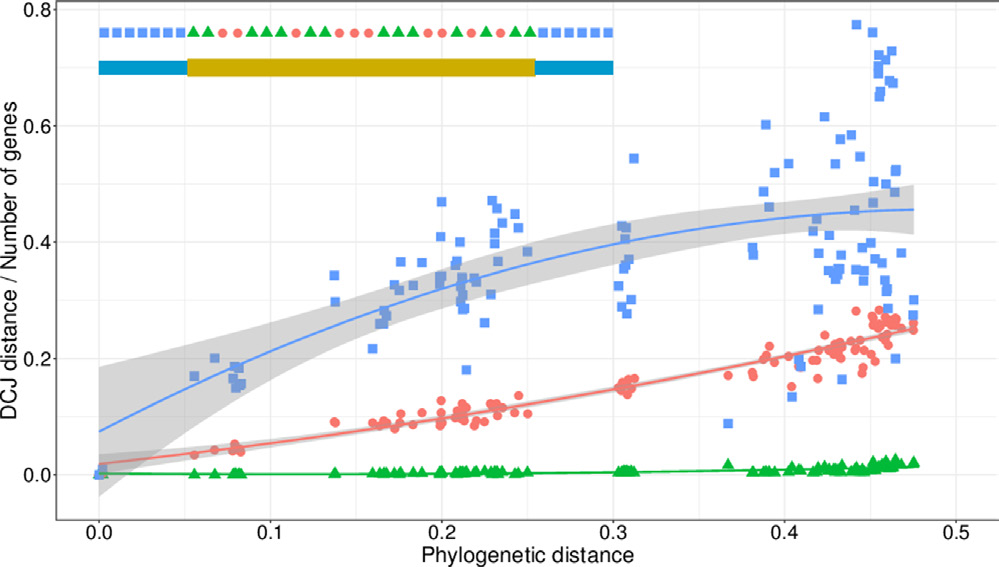
Propensity to rearrangements of *S. ambofaciens* ATCC 23877 genes. Three categories of genes have been defined for *S. ambofaciens* ATCC 23877 according to their location and level of conservation with other species: core-genes (green triangles), orthologous genes outside the core-region (blue squares) and orthologous genes inside the core-region (red dots). For each category, a weighted version of the DCJ [39 ) was calculated in pairwise comparisons and plotted against the phylogenetic distance between *S. ambofaciens* ATCC 23877 and the compared strains. A regression (polynomial degree 2) was applied, the grey zone is the 95 % confidence level interval for predictions from the model.

We further assessed the conservation of chromosome organization by applying the DCJ method [38], which infers a distance between two chromosomes based on the number of DNA rearrangement events. This distance can be estimated by the minimal number of shuffling events (inversion, transposition) needed to transform a given chromosome to the reference chromosome. This analysis was carried out from three different ways, distinguishing three categories of genes: the core-genes occupying by definition the core-region (i), the ‘non-core’ orthologues (i.e. orthologous genes excluding core-genes) either localized in the core-region (ii) or in the chromosomal arms (iii) as schematized in Fig. 5a. By plotting the DCJ distance against the phylogenetic distance between *S. ambofaciens* as a reference and all the other chromosomes (Fig. 5b), it appeared that these three gene sets behaved differently. The orthologues from the chromosomal arms displayed the most intense rearrangement pattern, which is fully consistent with the previous observations (i.e. GOC and NOC analyses). Focusing on the core-region, the trend of the core and the ‘non-core’ orthologues surprisingly showed distinct behaviours. The organization of the core-genes appears to be much more stable across evolutionary times than the non-core-genes of the core-region. This unexpected observation raises the hypothesis that the genes vertically inherited from the distant common ancestor (i.e. core-genes are most likely inherited from the ancestor of the genus) occupy crucial coordinates along the chromosome. In contrast, the genes which have been acquired (or lost) more recently, at different evolutionary times since the appearance of the genus, seem to occupy more permissive positions. In other words, ancestral genes appeared to be maintained at stable positions of the chromosome, and this could reflect a conserved chromosome skeleton.

### Chromosomal arms are fast evolving regions

**Fig. 6.**
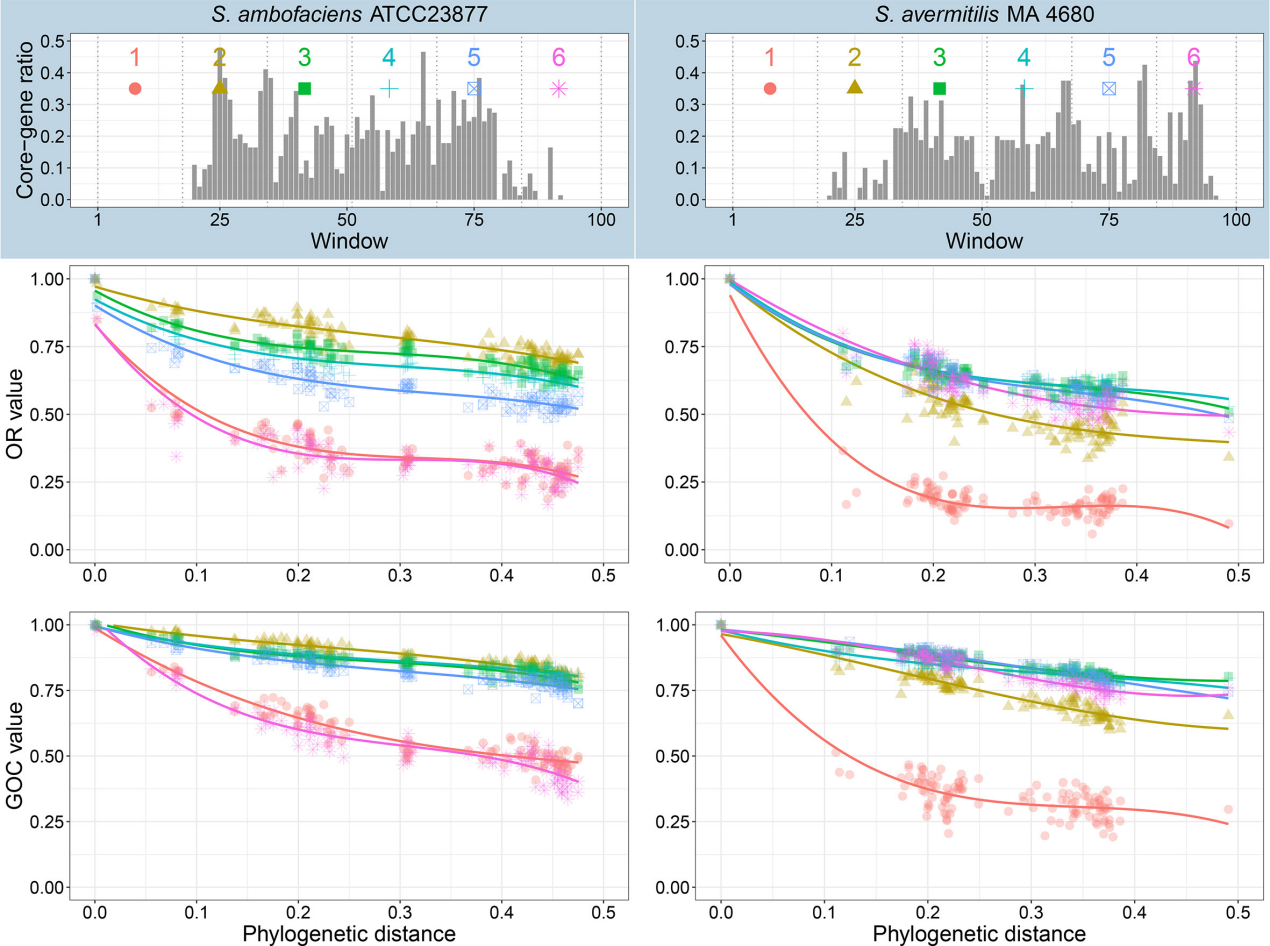
*
Streptomyces* subtelomeres are fast-evolving regions. Two reference species, *S. ambofaciens* ATCC 23877 and *S. avermitilis* MA-4680 were compared using OR and GOC indexes to the other species of our sample. The chromosome of each representative species was split into six parts containing the same number of genes (1235 and 1339, respectively, for *S. ambofaciens* ATCC 23877 and *S. avermitilis* MA-4680). The OR and GOC values for these six chromosomal parts were calculated in pairwise comparisons and plotted against the phylogenetic distance between the reference and the compared strains. A regression (polynomial degree 3) was applied. The upper panel gives the distribution of the core-genes along the considered chromosome with 100 adjacent windows equivalent in number of genes.

In order to better characterize the decline of synteny along the chromosomal arms, the chromosome used as a reference was split in portions equivalent by the number of genes and OR and GOC values were plotted against the phylogenetic distance through as many pairwise comparisons as possible in our dataset (124). Different tests, by dividing the genome from 6 to 100 pieces of equivalent size, were carried out in order to visualize the behaviours that could typify the different chromosomal regions regarding their evolutionary speed. Dividing the genome into 100 small fragments had the advantage of precision but increased the amount of data to be displayed. In fact, this attempt gave too many overlapping curves and finally illegible results (not shown). In contrast, cutting the genome into 6, 10 or 25 portions limited the amount of curves to be displayed (Figs 6, S6 and S7) but resulted in observing the trend of the index over large portions of the chromosome and therefore averaging the trends. Ultimately, such a cutout could prevent distinguishing the peculiar behaviour of some limited chromosomal areas (i.e. significantly smaller than the portion under consideration). In fact, it appeared that, in most cases, a six-piece cutout is sufficient to reveal the differential behaviour of the chromosomal parts.

Thus, as shown on Fig. 6, dividing the *S. ambofaciens* chromosome, which possesses a ‘typical’ chromosome (i.e. with two arms flanking the central region), into six pieces gave clear results and revealed compelling different evolutionary patterns: the two terminal portions (portions 1 and 6) showed drastically lower values for both GOC and OR than the rest of the chromosome. This reflects that these chromosomal regions are prone to a higher intensity of gene fluxes and recombination than the central part of the chromosome. Further, the decline of GOC and OR was more rapid for the two terminal regions than for the rest of the chromosome from the abscissa origin (i.e. from the closest pairs of species). Hence, in the distance range 0–0.1, the portions 1 and 6 showed a more significant OR decline (curve slope α of −5) than the portions 2–5, which showed a slope of −2 (Fig. 6). Frequent acquisition (horizontal gene transfer) and loss (recombination) of genetic material may rapidly renew and diversify the accessory genes mostly concentrated in the chromosomal arms. Therefore, it is no longer possible to interpret a differential evolution speed of the arms versus the central region. At great phylogenetic distance, this rapid renewal can lead to a saturation of this index in these regions. In other words, once the synteny is erased by the intense gene fluxes (reflected by very low OR indexes), further genetic exchange will no longer be detected. Consequently, it is no longer possible to interpret a differential speed of evolution of the arms versus the central region.

Interestingly, when cut into 25 portions (Fig. S7), certain regions (for example regions 3 and 4 on the left arm) show a behaviour that changes with the phylogenetic distance: a reminiscent behaviour of the phenomenon of degenerated synteny described by Choulet *et al*. [17] corresponding to conserved regions (high GOC and OR values) at short phylogenetic distance but progressively accumulating events of synteny rupture (decrease in GOC and OR values) with the increase of phylogenetic distance.

In contrast to *S. ambofaciens* where the chromosome has a ‘typical’ organization, *S. avermitilis* MA-4680 possesses an unbalanced chromosome: its left arm being at least five times the size of the right arm, as revealed by the distribution of the core-genes (Fig. 6). When the chromosome is cut into ten portions, two of them (numbered 1 and 2) showed significantly lower values of OR (Fig. S6) and span the left terminal region of the chromosome. Similarly, when the chromosome is cut into 25 portions (Fig. S7), seven of them (numbered 1, 2, 3, 4, 5, 6 and 25) showed low OR values. Portions 1–6 corresponded to the left arm while the portion 25 corresponded to the right one. The six-piece cutout revealed the same trend: only portion 1 corresponding to the left arm showed a rapid and strong decline for both GOC and OR indexes (Fig. 6). Therefore, it seems that the unbalanced chromosome of *S. avermitilis* MA-4680 also displayed an asymmetrical evolution pattern, consistent with the faster evolution of the arms compared to the core-region.

## Discussion

The results presented here show that the greater flexibility of the arms compared to that of the central region, a phenomenon initially suggested by Choulet *et al*. [17] on a sample of 3 genomes, is a general trend observed in the whole *Streptomyces* genus.

### The *Streptomyces* chromosomal arms are highly flexible

The arm plasticity we observed in this study is consistent with spontaneous chromosomal arm rearrangements observed under laboratory conditions: large deletions, amplifications or circularizations appear at high frequencies in the wild-type strain progeny [42–44]. These rearrangements are notably the result of the repair of double-stranded breaks (DSBs) either by HR or non homologous end joining (NHEJ), an IR mechanism recently characterized in *Streptomyces* [9, 45]. Indeed, artificially induced DSBs, particularly in the terminal regions, trigger all genomic rearrangements that appear spontaneously [9].

DSBs repair by HR or NHEJ can also promote drastic modifications of the terminal inverted repeats (TIR) size as observed at the genus level (Table S1) or even within the same population [24, 46, 47]. Recombination events between right and left arms of the same chromosome or of two newly replicated chromosomes alter both content and size of the TIR [9, 24]. Moreover, terminal recombination also occurs between replicons as evidenced by the formation of hybrid chromosomes and plasmids in *S. coelicolor* [48] or *Streptomyces rimosus* [49]. This inter-replicon recombination mechanism is likely to provoke, through a single mutational event, the erasing of the ancestral synteny, and to generate evolutionary jumps in the genetic diversification of *Streptomyces* chromosome. Altogether, these rearrangements could be responsible for some of the terminal diversity found within the genus and explain the formation of ‘unbalanced’ chromosomes, i.e. with chromosome arms that are not equal in size.

### Identification of a chromosomal skeleton

Our data refine the accepted view [14, 18] of a compartmentalized *Streptomyces* chromosome with unstable arms framing a more constrained central region by highlighting that the central region is not a homogeneous block but consists of a skeleton formed in particular by the genes of the core-genome, between which a certain plasticity is tolerated. The architectural constraints imposed on this skeleton may be related to main cellular processes such as chromosomal replication or *in vivo* three-dimensional chromosome organization, which is correlated to gene expression and cell division [5, 50–53]. Replication is a major structuring force of the bacterial chromosome. The two replichores, i.e. the two halves of the chromosome defined by the position of the origin of replication (*oriC*) and the opposite terminus, have a symmetrical organization, both in terms of nucleotide composition and gene organization. In bacteria, a marked strand-biassed distribution of the genes was observed between the replicating strand. This bias was shown to be driven by gene essentiality by Rocha and Danchin [54]. In *Streptomyces *, where the central position of *oriC*, defines two replichores with natural termini corresponding to the telomeres, the strand-bias is also found since in *S. ambofaciens* ATCC 23877 chromosome, about 71 % of the core-genes showed a transcriptional orientation in the direction of the continuous replication versus only 57 % for the rest of the genes (not shown). This observation is coherent since the core-genome is likely to be enriched in essential genes. The preferential distribution of the core-genes in the central part of the linear chromosome is also consistent with a stronger expression of these genes. Hence genes located close to the origin have a higher copy number per cell due to replication reinitiation (the so-called gene-dosage effect), and can therefore show a stronger expression in active growth than in stationary phase. In a global transcriptome analysis of *S. ambofaciens* ATCC 23877, that the majority of the 1018 core-genes (55 %) was shown to belong to the most expressed genes in at least one of the growth conditions tested (S. Bury-Moné, personal communication). The most expressed genes represent only 25.8 % of the total predicted genes (7084 CDSs). Therefore, the 1018 core-genes whose organization appeared particularly conserved could constitute such a chromosomal skeleton.

Chromosomal partition and its coupling with cell division is also a structuring force. Thus, the macrodomains Ori and Ter [55], which represent about 1 Mb each in *E. coli* [56] are positioned in the cells in such a way that they promote an equitable and efficient segregation of the newly replicated chromosomes in the daughter cells. The choreography of the chromosomes is regulated by the condensation of these domains and their interaction with the actors of cell division [57]. The perturbation of these domains, notably by chromosomal rearrangements such as translocations and inversions [58], is strongly counter-selected, revealing the constraint of chromosome organization. The three-dimensional structuring of the genome is also linked to global gene expression. Thus, in prokaryotes as in eukaryotes where this phenomenon was first identified, DNA folding is strongly influenced by the position of highly expressed genes, the latter forming the boundaries of folding domains (CID, TAD [59]). Hence, the positioning of core-genes could determine the structuring of the nucleoid *in vivo* and could contribute to the course of cell replication/segregation/division processes. Consequently, the organization of these genes or loci could constitute a strong evolutionary constraint.

### What mechanism(s) could account for *Streptomyces* chromosomal organization?

**Fig. 7.**
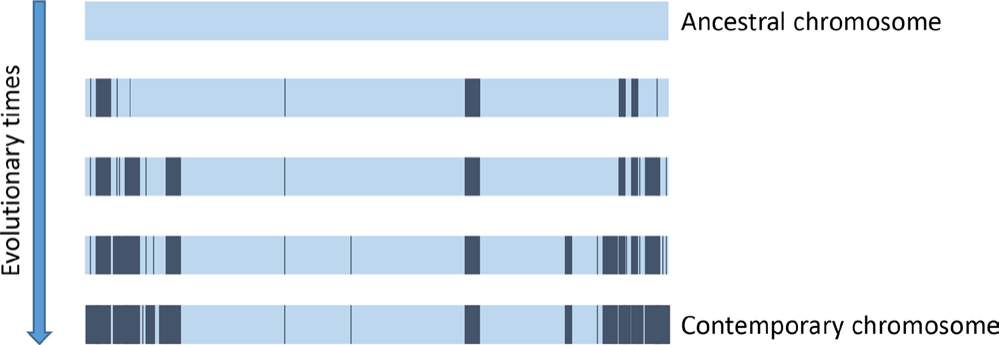
Model of *Streptomyces* chromosome evolution by accumulation of indels in the terminal regions. Preferential accumulation of indels according to a recombination gradient in the terminal regions of the chromosome is schematized. Accumulation over evolutionary time increases terminal diversification while maintaining a rather conserved central region. Strains from the same common ancestor diverge more in their terminal regions that have become specific than in the region containing the conserved genes (core-genes).

Altogether, our results allow us to propose a global hypothesis about the mechanisms shaping *Streptomyces* chromosome where both gene flux and shuffling resulting from recombination activities are operating more intensively in the arms than in the central part. These results also point out the progressive decline of synteny all along the arms, towards the chromosomal ends. This recombination gradient had already been reported, thanks to the comparison of a few complete genome sequences [17]. This observation suggested that diversification occurred mainly by the cumulation of recombination events through evolutionary times (Fig. 7). Recently, genome comparison within a natural population isolated from the same micro-niche at the millimetre scale [24] gave consistent conclusions: although having diverged over a short evolutionary time, *Streptomyces* strains showed a significant amount of genomic differences consisting of insertions and deletions (indels) preferentially located in the chromosomal arms. A close analysis revealed that, even at this phylogenetic distance, the indels' distribution formed a gradient increasing in intensity towards the chromosomal ends. This picture may result from the presence of an increasing recombination gradient towards the chromosomal extremities; a recombination gradient on which selective phenomena are superimposed.

Therefore, we propose, as schematized on Fig. 7, that the accumulation of indels according to this recombination gradient accompany the divergence of strains from their common ancestor. The recombinogenic activity in the chromosomal arms may be stimulated by the more frequent formation of DSBs in the subtelomeric regions of the chromosome. Indeed, DSBs could result from arrests or slow-downs of the replication fork [60, 61], which provide natural replication termination regions and that would be more frequent in subtelomeres. Another source of terminal breaks could be the accidental guillotining of the daughter chromatids by septal closure, notably during sporulation [62]. The gradient may also result from a differential efficiency of DSB repair systems along the genome. This spatial regulation of repair systems was described in yeast where DSB are more efficiently repaired through HR when occurring in the telomere regions [63] while IR (i.e. NHEJ) keeps the same efficiency along the chromosome. In eukaryotes [64–66], recent studies emphasize the importance of the modulation of chromosomal architecture in the control of recombination. Interestingly, the *Streptomyces* terminal regions form specific chromatin domains in configuration chromosome capture (3C) and show a loose structuration compared to the rest of the chromosome (P. Leblond, M. Marbouty and R. Koszul, personal communication). The links between chromatin structure and recombination in bacteria have yet to be explored.

### Chromosome arms: hot spots for evolving social genes

The high recombinogenic activity observed in the chromosomal arms maintains a strong selection pressure on the genes present in these regions [5]. Thus, genes of little adaptive value (and *a fortiori* pseudogenes) can be easily eliminated in favour of genes contributing to a better fitness. We can thus speculate that the distribution of genes along the chromosome could be correlated to their respective contribution to the biology of the bacterium. Hence, genes producing ‘private goods’, meaning providing a benefit only to the individual cell that produced them, such as genes involved in the vegetative cycle, should therefore be maintained and transmitted to the offspring. Their positioning in the central part of the chromosome could have been selected for since their presence in the arms would have exposed them to short-term deleterious effects of DNA rearrangements.

Bacteria also produce extracellular molecules that can be considered as ‘public goods’ (e.g. extracellular enzymes or metabolites such as antibiotics and antifungals) since they can potentially benefit other cells and establish social traits [67]. The ‘social genes’, could be expressed by only a part of the population for the benefit of all. The selective pressure on these genes would apply to the population as a whole and not at the individual cell scale where the pressure would then be weaker. We speculate that, in *Streptomyces *, the positioning of these social genes in the flexible arms would be selected for. Therefore, the arm diversity, by allowing the population to benefit from a metabolic skill pool much broader than an individual bacterial cell, would confer an advantage at the populational level. For instance, the case of the biosynthetic gene clusters (BGCs) involved in the production of a wide variety of compounds including antibiotics illustrates this concept; the terminal regions are enriched in these kinds of genes compared to the central region of the chromosome [13]. BGCs are indeed highly variable between species and even between strains of the same population probably reflecting their strong adaptive value in some specific circumstances such as biotic competition [13]. Their terminal localization can also favour their replacement by incoming BGCs ensuring the rapid diversification of the metabolite reservoir.

For decades, the evolutionary impact of genomic instability has been in question, as the formation of mutants at high frequencies (at least 1%, [68, 69]) with rearranged chromosomes and consequently deficient in differentiation traits (such as sporulation, colony pigmentation, antibiotic synthesis) could have been interpreted as an evolutionary dead end or even as a laboratory artefact. If such rearrangements may indeed be deleterious at the individual level, recent evidence showed that this genomic heterogeneity may participate to a better adaptability of a whole population [24, 70]. Hence, Daniel Rozen and coworkers have shown that a co-culture containing mutant (harbouring DNA amplifications and/or deletions affecting the chromosomal arms) and wild-type cells of *S. coelicolor* A3(2) was more efficient than the wild-type alone to produce antibiotics. Individually, these mutants sporulate less efficiently than the wild-type strain, but synthesize more antibiotics. Therefore, it can be assumed that collectively each member of a population ensures a specific task, establishing a division of labour between members of the same population [24, 70].

## Supplementary Data

Supplementary material 1
