# Supplementary material for: Subtelomeres are fast-evolving regions of the Streptomyces linear chromosome
Source: Microb Genom. 2021 Mar 22;7(6):000525. doi: 10.1099/mgen.0.000525 (PMC8627663; doi:10.1099/mgen.0.000525)
Supplement: Supplementary material 1 [file mgen-7-0525-s001.pdf]

Supplementary files

Subtelomeres are fast-evolving regions of the *Streptomyces* linear chromosome

Jean-Noël Lorenzi[1,2], Olivier Lespinet[1*], Pierre Leblond[2*], Annabelle Thibessard[2*]

[1]Université Paris-Saclay, CEA, CNRS, Institute for Integrative Biology of the Cell (I2BC), 91198, Gif-sur-Yvette, France.

[2]Université de Lorraine, INRA, DynAMic, F-54000 Nancy, France

Table 1: List of the *Streptomyces* species studied in this work.

Name	Genome Assembly ID	Chromosome length (bp)	Plasmid length (bp)	CDSs nb	TIR length (bp)
Streptomyces sp. S1A1 7	ASM711356v2	11,713,151	292,353 [1]	11,068 [265]	ND
S. bingchenggensis BCW 1	ASM9238v1	11,936,683	-	10,216	ND
Streptomyces sp. 11 1 2	ASM359554v1	11,603,877	51,329 [1]	9,986 [61]	29,227
Streptomyces sp. GGCR 6	ASM395571v1	10,393,987	-	9,540	96,011
Streptomyces sp. CdTB01	ASM148456v1	9,902,731	288,836 [1]	9,444 [287]	400,291
Streptomyces sp. YIM 121038	ASM608871v1	10,130,554	967,890 [1]	9,440 [840]	128,054
S. hygroscopicus jinggangensis 5008	ASM24535v1	10,145,833	237,851 [2]	9,401 [243]	ND
Streptomyces sp. GY16	ASM918486v1	10,116,136	59,304 [1]	9,248 [66]	ND
Streptomyces sp. 769	ASM81602v1	10,100,774	237,512 [1]	9,180 [224]	155,968
Streptomyces sp. T44	ASM979628v1	9,735,779	-	9,126	ND
S. scabiei 87 22	ASM9130v1	10,148,695	-	9,009	18,488
Streptomyces sp. P3	ASM303247v1	9,851,971	-	8,820	ND
S. noursei ATCC 11455	ASM170427v1	9,815,884	-	8,811	170,209
S. kanamyceticus ATCC 12853	ASM870449v1	10,133,897	-	8,806	ND
S. chartreusis ATCC 14922	ASM870471v1	9,912,098	-	8,704	ND
S. atratus SCSIO ZH16	ASM333086v1	9,641,288	-	8,700	288,246
S. lincolnensis LC G	ASM334444v1	9,513,637	-	8,622	ND
S. griseorubiginosus 3E 1	ASM359523v1	9,512,378	-	8,551	113,251
S. lydicus A02	ASM95203v2	9,307,519	-	8,534	52,505
Streptomyces sp. CC0208	ASM344373v1	9,320,089	-	8,531	ND
S. coeruleorubidus ATCC 13740	ASM870513v1	9,335,698	-	8,518	ND
Streptomyces sp. NEAU S7GS2	ASM317327v1	9,641,634	45,805 [1]	8,508 [52]	ND
S. ectabilis ATCC 27465	ASM870479v1	9,807,160	-	8,329	112,273
S. coelicolor A3 2	ASM20383v1	8,667,507	387,340 [2]	8,320 [400]	21,653
S. formicae KY5	ASM255654v1	9,611,874	-	8,317	35,482
S. vietnamensis GIM4 0001	ASM83000v1	8,867,142	286,635 [1]	8,287 [271]	49,821
S. rimosus ATCC 10970	ASM870465v1	9,361,154	-	8,287	11,287
S. autolyticus CGMCC0516	ASM198397v1	10,029,028	155,632 [7]	8,279 [36]	ND
S. griseorubiginosus BTU6	ASM651693v1	9,226,027	-	8,274	224,191
S. albulus CK 15	ASM93518v3	9,336,218	-	8,241	ND
Streptomyces sp. S10 2016	ASM161179v1	9,083,372	-	8,241	134,209
S. dengpaensis XZHG99	ASM294683v1	8,541,354	168,817 [2]	8,236 [210]	21,003
Streptomyces sp. ADI95 16	ASM372149v1	8,184,000	902,421 [4]	8,223 [861]	ND
S. avermitilis MA 4680	ASM976v2	9,025,608	94,287 [1]	8,132 [94]	ND
S. lavendulae CCM 3239	ASM280384v1	8,691,711	241,081 [1]	8,085 [231]	237,734
S. chartreusis NRRL 3882	NRRL3882	8,983,317	-	8,083	39,831
S. ectabilis NRRL 2792	ASM736339v1	9,505,665	-	8,055	ND
Streptomyces sp. M2	ASM410450v1	8,718,751	-	8,025	ND
Streptomyces sp. SYP A7193	ASM949565v1	8,193,527	629,255 [2]	7,984 [571]	ND
S. lydicus WYEC 108	ASM399437v1	9,125,666	-	7,970	8,910
S. venezuelae ATCC 14584	ASM864231v1	8,942,078	-	7,935	165,025
S. lunaelactis MM109	ASM305455v1	8,396,100	174,091 [2]	7,873 [149]	ND
Streptomyces sp. Mg1	ASM41226v2	7,868,178	848,015 [3]	7,864 [812]	ND
Streptomyces sp. QMT 28	ASM949827v1	8,880,330	-	7,861	ND
S. griseoviridis F1 27	ASM399439v1	8,963,414	-	7,785	58,707
S. venezuelae ATCC 15068	ASM864237v1	8,558,202	-	7,743	ND
Streptomyces sp. Go 475	ASM333084v1	8,570,609	-	7,729	6,902
S. gilvosporeus F607	ASM208219v1	8,482,298	-	7,624	ND
S. olivoreticuli ATCC 31159	ASM339113v1	8,809,793	-	7,593	170,471
S. hundungensis BH38	ASM362781v1	8,393,044	-	7,541	65,122
S. ambofaciens ATCC 23877	ASM126788v1	8,303,940	89,658 [1]	7,541 [128]	202,695
S. alfalfae ACCC40021	ASM197502v1	8,625,867	-	7,525	149,241
Streptomyces sp. SUK 48	ASM965076v1	8,341,671	-	7,507	188,470
Streptomyces sp. CCM MD2014	ASM77204v1	8,274,043	-	7,501	14,382
S. pristinaespiralis HCCB 10218	ASM127807v1	8,532,592	-	7,498	419,828
S. platensis ATCC 23948	ASM870485v1	8,501,012	-	7,490	24,984

(continued on next page)

Table 1: List of the *Streptomyces* species studied in this work.

Name	Genome Assembly ID	Chromosome length (bp)	Plasmid length (bp)	CDSs nb	TIR length (bp)
S. rochei 7434AN4	ASM806499v1	8,364,802	-	7,485	53,905
Streptomyces sp. W1SF4	ASM395003v1	7,272,878	795,265 [2]	7,420 [656]	ND
S. actuosus ATCC 25421	ASM320803v1	8,145,579	-	7,399	17,181
S. endophyte N2	ASM410448v1	8,428,700	-	7,398	53,557
Streptomyces sp. Sge12	ASM208045v1	7,983,613	127,085 [1]	7,376 [119]	162,199
S. pactum ACT12	ASM200522v1	8,550,793	-	7,375	239,610
Streptomyces sp. WAC 01438	ASM394552v1	8,138,328	62,839 [1]	7,350 [72]	49,769
S. lydicus GS93 23	ASM198444v1	8,243,179	-	7,320	36,373
S. griseus NBRC 13350	ASM1060v1	8,545,929	-	7,306	132,910
S. venezuelae ATCC 14583	ASM864235v1	8,018,484	-	7,301	157,265
Streptomyces sp. SS52	ASM479571v1	8,184,045	-	7,293	43,391
S. collinus Tu 365	ASM44487v1	8,272,925	104,361 [2]	7,283 [101]	631,364
Streptomyces sp. WAC 01529	ASM394554v1	8,270,461	-	7,269	58,922
Streptomyces sp. KPB2	ASM395005v1	8,082,236	-	7,256	40,616
S. ambofaciens DSM 40697	ASM163286v1	8,137,876	-	7,250	212,696
Streptomyces sp. fd1 xmd	ASM200768v1	7,929,999	-	7,236	ND
S. clavuligerus ATCC 27064 2 3	ASM551946v1	6,748,591	1,795,495 [1]	7,226 [1,534]	ND
Streptomyces sp. GSSD 12	ASM334496v1	8,454,852	-	7,201	ND
S. katrae S3	ASM202842v1	7,504,851	551,599 [2]	7,196 [321]	ND
Streptomyces sp. CFMR 7 CFMR 7	ASM127809v1	8,207,742	99,537 [1]	7,190 [95]	11,739
S. cattleya NRRL 8057	ASM23730v1	6,283,062	1,809,491 [1]	7,189 [1,651]	ND
S. tsukubensis AT3	ASM929602v1	8,615,214	18,766 [1]	7,183 [13]	17,935
S. venezuelae ATCC 14585	ASM864233v1	8,048,154	-	7,176	166,199
S. leeuwenhoekii sleC34	sleC34	7,903,895	218,596 [2]	7,120 [263]	388,272
Streptomyces sp. QHH 9511	ASM978963v1	7,524,079	91,197 [1]	7,109 [124]	ND
S. niveus SCSIO 3406	ASM200917v1	7,990,492	-	7,105	ND
Streptomyces sp. CB09001	ASM336979v1	7,787,608	-	7,085	ND
S. globisporus C 1027	ASM26134v2	7,608,611	174,988 [2]	7,079 [154]	ND
S. fulvissimus DSM 40593	ASM38594v1	7,905,758	-	7,072	ND
S. albireticuli MDJK11	ASM219245v1	8,144,417	-	7,024	133,288
S. vinaceus ATCC 27476	ASM870493v1	7,673,509	-	7,020	ND
S. venezuelae ATCC 10595	ASM870525v1	7,871,480	-	7,001	ND
S. galilaeus ATCC 14969	ASM870457v1	7,756,194	-	7,001	ND
Streptomyces sp. Tue6075	ASM193163v1	7,931,832	-	6,994	12,088
S. alboniger ATCC 12461	ASM870439v1	7,962,786	-	6,993	ND
S. nigra 452	ASM307405v1	7,641,029	-	6,992	107,944
S. violaceoruber S21	ASM208217v1	7,916,045	-	6,979	4,122
S. parvulus 2297	ASM166004v1	7,149,446	617,085 [1]	6,963 [443]	ND
S. bacillaris ATCC 15855	ASM326867v1	7,888,441	-	6,953	ND
Streptomyces sp. TN58	ASM194184v1	7,585,034	-	6,936	193,939
S. venezuelae ATCC 21018	ASM864227v1	7,746,267	-	6,919	ND
S. nodosus ATCC 14899 2	ASM870499v1	7,772,587	-	6,917	ND
S. albus ZD11	ASM367534v1	8,317,371	-	6,904	614,456
S. subrutilus ATCC 27467	ASM870453v1	7,604,974	-	6,839	162,000
S. fungicidicus TXX3120	ASM38594v1	6,740,768	926,728 [1]	6,839 [841]	ND
S. tsukubensis NRRL 18488	ASM393271v1	7,963,742	55,806 [2]	6,809 [63]	210,577
S. cinereoruber ATCC 19740	ASM929938v1	7,516,652	-	6,807	ND
S. prasinus ATCC 13879	ASM870444v1	7,647,592	-	6,793	ND
Streptomyces sp. SM18	ASM291077v2	7,703,166	-	6,783	14,611
Streptomyces sp. PAMC26508	ASM36480v1	7,526,197	104,048 [1]	6,779 [84]	36,502
S. nitrosporeus ATCC 12769	ASM870455v1	7,581,562	-	6,778	26,990
Streptomyces sp. SSL 25	ASM785615v1	8,146,484	-	6,768	132,197
S. glaucescens GLA O	ASM76121v1	7,453,200	170,574 [1]	6,715 [140]	14,128
S. asterosporus DSM 41452	ASM671613v1	7,766,581	-	6,710	ND
S. venezuelae ATCC 21782	ASM864229v1	7,525,322	-	6,699	182,221

(continued on next page)

Name	Genome Assembly ID	Chromosome length (bp)	Plasmid length (bp)	CDSs nb	TIR length (bp)
S. cyanogriseus noncyanogenus NMWT 1	ASM93144v1	7,762,396	-	6,681	ND
Streptomyces sp. SirexAA E	ASM17719v2	7,414,440	-	6,663	ND
Streptomyces sp. S8	ASM209499v1	7,529,075	72,789 [1]	6,624 [78]	12,184
S. viridosporus T7A ATCC 39115	ASM870451v1	7,280,536	-	6,603	ND
S. luteoverticillatus CGMCC 15060	ASM397071v1	7,367,863	-	6,574	ND
S. pluripotens MUSC 135	ASM80224v2	7,346,075	-	6,561	ND
Streptomyces sp. SAT1	ASM165449v1	7,472,530	-	6,503	ND
S. ficellus NRRL 8067	ASM973990v1	7,078,240	-	6,412	21,921
S. koyangensis VK A60T	ASM342892v1	7,220,839	-	6,365	20,855
S. tirandamycinicus HNM0039	ASM309751v1	7,289,495	-	6,363	ND
S. albidoflavus SM254	ASM157738v1	7,170,504	-	6,298	ND
S. ongiicola HNM0071	ASM312236v1	7,180,417	-	6,166	ND
S. fradiae ATCC 10745	ASM870442v1	6,725,579	-	5,877	48,424
S. seoulensis KCTC 9819	ASM432862v1	6,339,363	86,882 [1]	5,866 [107]	20,875

Name: species name; Genome Assembly ID: unique identifier used in the NCBI Genome Assembly database; Chromosome length (bp): chromosome length expressed in pb; Plasmid length: cumulative length of plasmids expressed in pb [plasmid number in the genome]; CDSs Nb : CDS number in the whole genome [CDS number in plasmids], the data was ordered according to this parameter in descending order; TIR length (bp): size of one copy of a TIR expressed in pb. The estimate of the TIR and chromosome sizes has been established according to the public data submitted to NCBI and the possible absence of the extremities may lead to a slight underestimation.

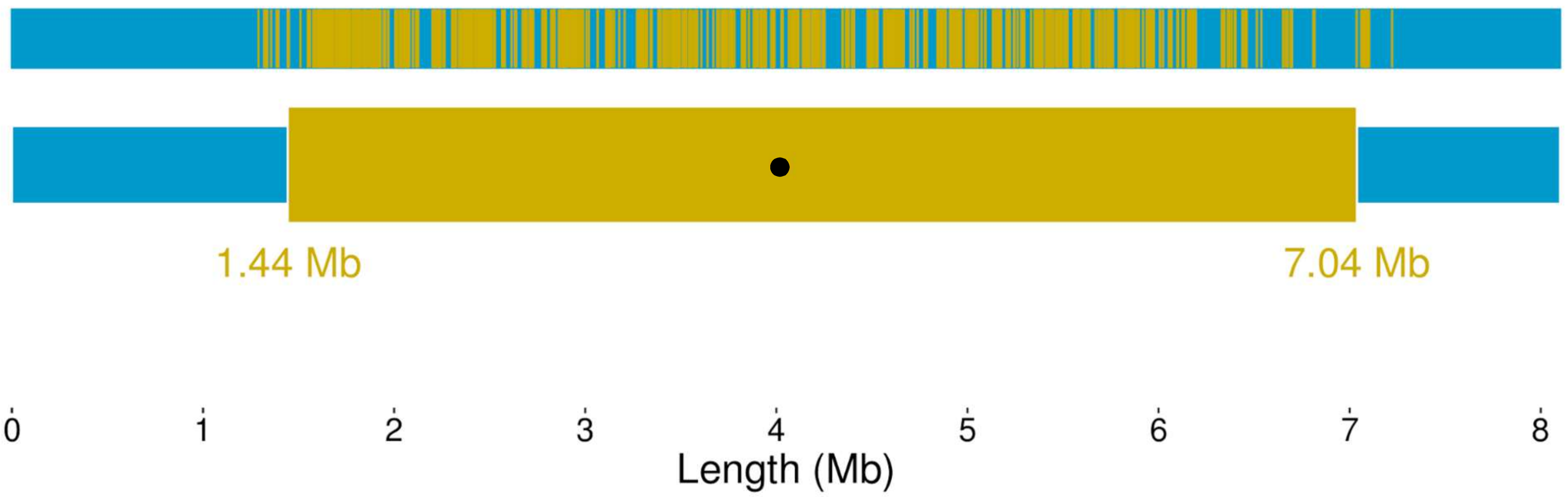

Figure S1: Schematic representation of *S. ambofaciens* ATCC 23877 chromosome.
The upper panel shows the distribution of core-genes (in ochre) along the chromosome of *S. ambofaciens* ATCC23877 and the lower panel displays the representation of this chromosome where the core-region is in ochre and the chromosome arms are in blue. The *oriC* locus is symbolized by a black dot. Numbers (Mb) represent the limits of the core-region.

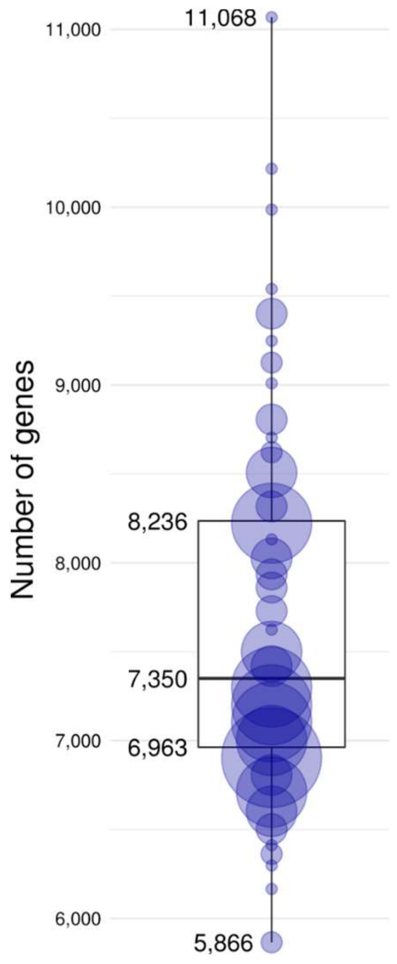

Figure S2: Genomes size distribution of the 125 *Streptomyces* species.
The lower and upper whiskers of the boxplot show the first quartile and the last quartile, the horizontal central line indicates the median value. The blue circles indicate the number of genomes in our data set where the radius of each circle is proportional to the number of genomes in this range of size.

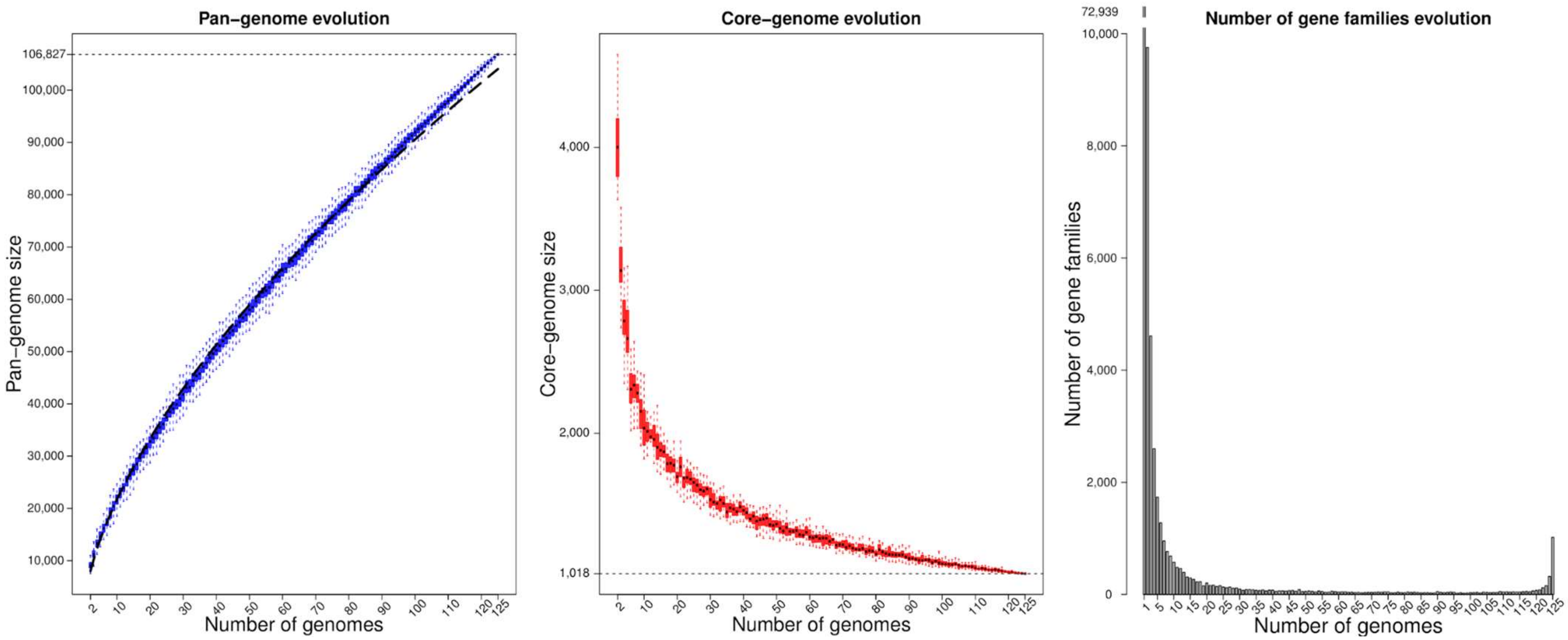

Figure S3: Pan-genome, core-genome and number of gene families evolution according to the number of genomes.
For a number of genomes varying from 2 to 125, the size of the pan-genome and core-genome was approached after 100 iterations of a random selection of genomes picked in our dataset. Each point is a whisker box with the lower and upper whiskers corresponding to the first and the last quartile of the distribution. The horizontal line gives the median value. The pan- and core-genomes were estimated to be 106,827 and 1,018 genes, respectively, for the whole set of species. On the pan-genome evolution graph, the the dotted black curve represents the least-square fit of the function $f(x) = Kx^{\gamma}$ to data (with $K = 5204.028$ and $\gamma = 0.6203757$).
The right panel shows the evolution of the number of gene families according to the number of genomes, varying from 1 to 125. The value obtained for one genome reaches the value of 72,939, and the histogram bar was broken to symbolize the non-continuity of the ordinate axis for this abscissa. This value shows that 68% of the pan-genome is composed of specific genes.

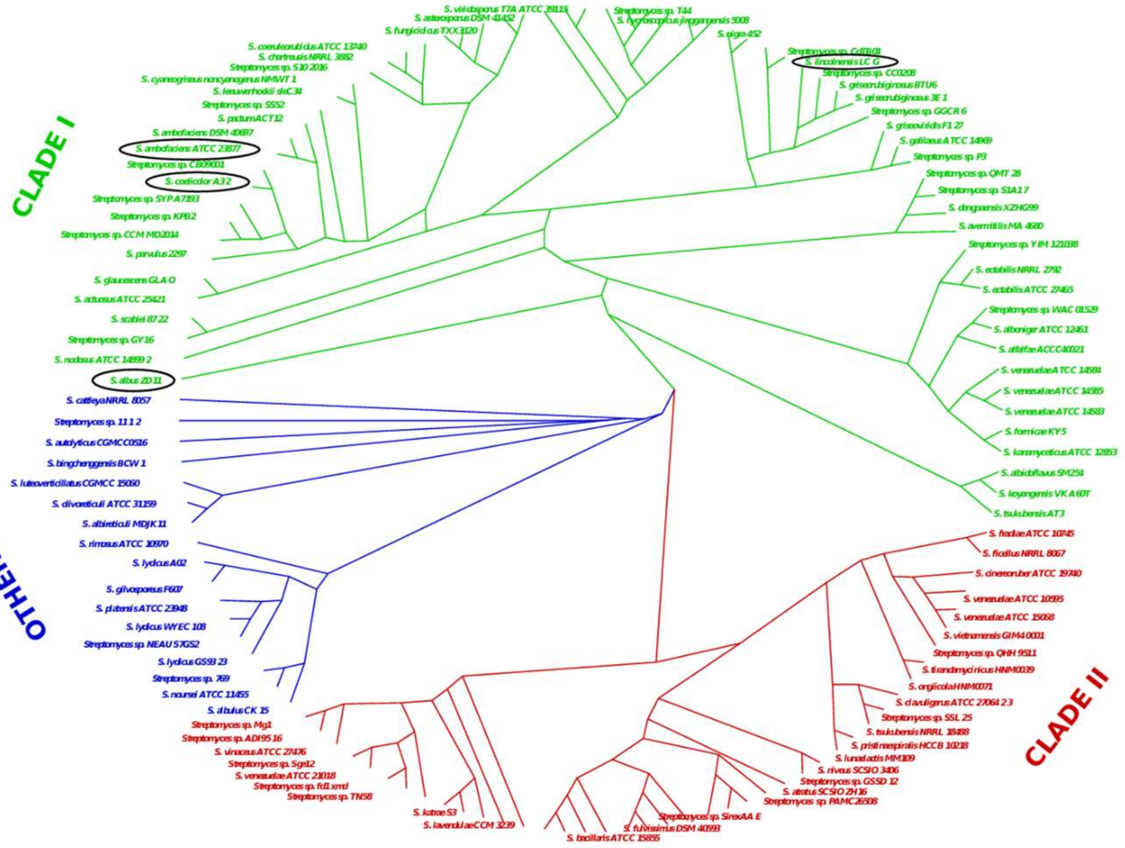

Figure S4: Phylogenetic tree of the 125 *Streptomyces* species.
The Maximum likelihood approach was used to reconstruct the phylogenetic relationships between our species/strains from the 1,018 genes of the *Streptomyces* core-genome. The different monophyletic clades identified and consistent with McDonald and Currie (2017) are indicated. The bootstrap values obtained after 100 bootstrap replications are all greater to 97% and are not shown. Species used in figure 3 are identified by a circle.

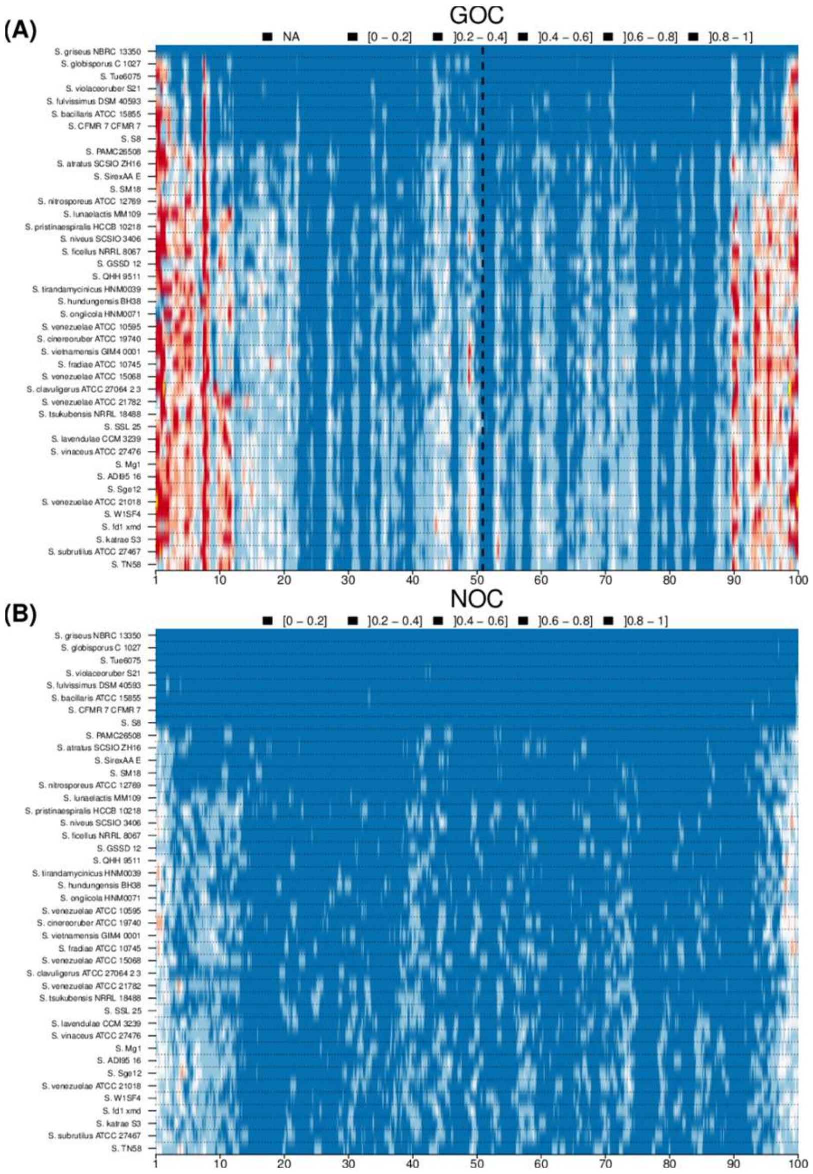

Figure S5: GOC and NOC trends along the *Streptomyces* chromosome.

The local GOC (**A**) and NOC (**B**) values are represented as a heatmap for the *Streptomyces* species belonging to clades I and using *S. griseus* NBRC 13350 as a reference.

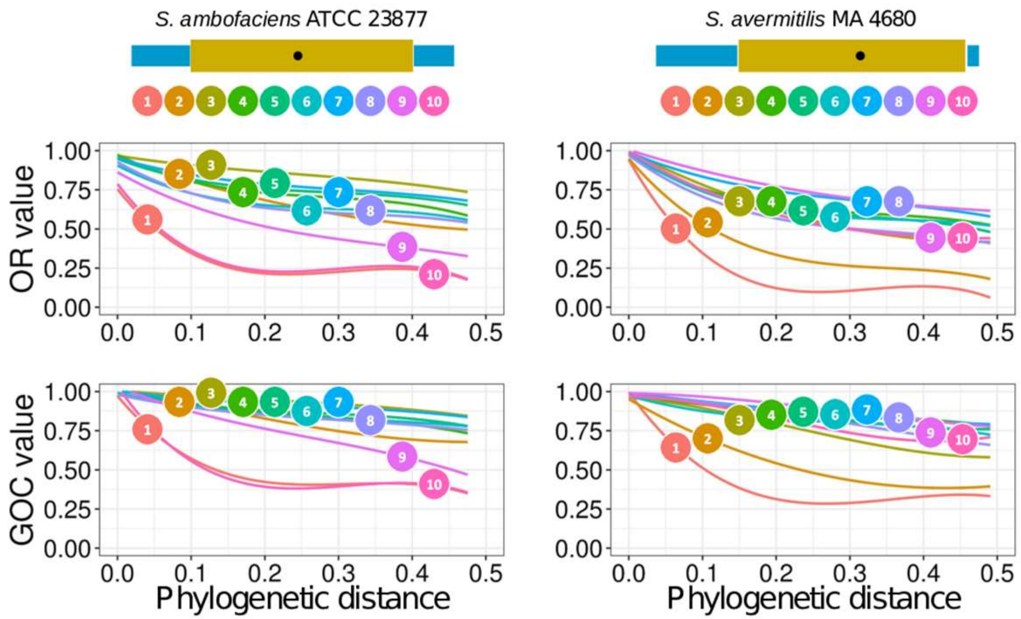

Figure S6: Survey of evolution speed of the *S. ambofaciens* ATCC 23877 and *S. avermitilis* MA-4680 SAM chromosomal regions by OR and GOC indexes. The OR and GOC values for the two chromosomes cut into 10 (containing each the same number of genes) were calculated in pairwise comparisons and plotted against the phylogenetic distance between the reference and the compared strains. The chromosome portions have been numbered as shown in the insert at the top of the figure. The numbers in the coloured tablets on the curves reflect this numbering and make it easier to identify the chromosome portions. A regression (polynomial degree 3) was applied to visualize the evolution speed of the OR and GOC indexes by chromosome portion.

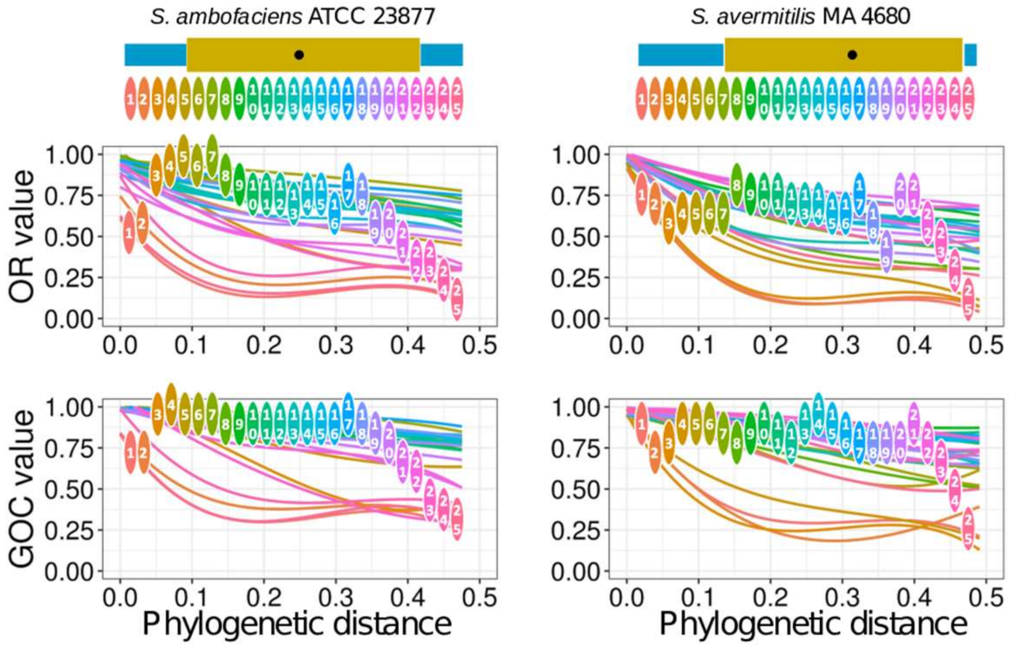

Figure S7: Survey of evolution speed of the *S. ambofaciens* ATCC 23877 and *S. avermitilis* MA-4680 SAM chromosomal regions by OR and GOC indexes. The OR and GOC values for the two chromosomes cut into 25 portions (containing each the same number of genes) were calculated in pairwise **comparisons** and plotted against the phylogenetic distance between the reference and the compared strains. The chromosome portions have been numbered as shown in the insert at the top of the figure. The numbers in the coloured tablets on the curves reflect this numbering and make it easier to identify the chromosome portions. A regression (polynomial degree 3) was applied to visualize the evolution speed of the OR and GOC indexes by chromosome portion.
